# A Novel Chitin Binding Crayfish Molar Tooth Protein with Elasticity Properties

**DOI:** 10.1371/journal.pone.0127871

**Published:** 2015-05-26

**Authors:** Jenny Tynyakov, Shmuel Bentov, Shai Abehsera, Isam Khalaila, Rivka Manor, Lihie Katzir Abilevich, Simy Weil, Eliahu D. Aflalo, Amir Sagi

**Affiliations:** 1 Department of Life Sciences, Ben-Gurion University, Beer-Sheva, Israel; 2 National Institute for Biotechnology in the Negev, Ben-Gurion University, Beer-Sheva, Israel; 3 Department of Biotechnology Engineering, Ben-Gurion University, Beer-Sheva, Israel; Uppsala University, SWEDEN

## Abstract

The molar tooth of the crayfish *Cherax quadricarinatus* is part of the mandible, and is covered by a layer of apatite (calcium phosphate). This tooth sheds and is regenerated during each molting cycle together with the rest of the exoskeleton. We discovered that molar calcification occurs at the pre-molt stage, unlike calcification of the rest of the new exoskeleton. We further identified a novel molar protein from *C*. *quadricarinatus* and cloned its transcript from the molar-forming epithelium. We termed this protein Cq-M13. The temporal level of transcription of *Cq-M13* in an NGS library of molar-forming epithelium at different molt stages coincides with the assembly and mineralization pattern of the molar tooth. The predicted protein was found to be related to the pro-resilin family of cuticular proteins. Functionally, *in vivo* silencing of the transcript caused molt cycle delay and a recombinant version of the protein was found to bind chitin and exhibited elastic properties.

## Introduction

The mandible of the crayfish *Cherax quadricarinatus* consists of a large base, capped with two protuberances: the molar and the incisor teeth. The molar tooth is comprised of two different zones, the apatite (calcium phosphate) mineral layer at its crown and chitin with amorphous minerals composed of calcium carbonate and calcium phosphate at its bulk [1]. The growth of crustaceans demands periodic shedding and replacement of the exoskeleton. A new exoskeleton is deposited during pre-molt and sclerotized and calcified during the post-molt stage [2]. Since the molar tooth is part of the exoskeleton it is periodically removed and regenerated during each molting cycle. The size of the gastroliths is used as a pre-molt indicator to monitor the molting cycle in the crayfish [3].

In vertebrates, various proteins have been suggested to play important roles in the mineralization of bones and teeth [4]. Similarly, in invertebrates, skeletal proteins also play crucial roles in mineral formation [5, 6], and several crustacean proteins thought to be associated with calcium carbonate calcification have been identified *in vitro* [7]. *In vivo* studies point to some of these proteins as influencing enamel plasticity and morphology, as well as the mineral content of teeth [8, 9]. Accordingly, mineralization experiments have revealed that recombinant versions of these proteins contribute to the regulation of the morphology and organization of mineral particles *in vitro* [10, 11].

In crustaceans, the mineral is precipitated on an extracellular chitinous scaffold. As such, many exoskeletal proteins were found to contain chitin-binding domains [12], such as chitin-binding domain 2 [13] and the Rebers—Riddiford (R-R) consensus sequence [14], and/or to interact with calcium carbonate. Three distinct forms of the R-R consensus sequences, RR-1, RR-2, and RR-3, have all been recognized in crustacean proteins [15] and were shown experimentally to bind chitin [16]. Still, much remains unknown regarding the different R-R sequence-containing proteins, such as their localization in the exoskeleton and the nature of the interaction between such proteins, chitin and minerals. In addition, some of the cuticle proteins possessing a chitin-binding R-R consensus sequence [16, 17], were assigned as resilin (a cuticular elastic protein) homologs.

Elastic proteins vary greatly and spread through all over animal kingdom, including humans [18]. Among their roles are material protection from a fatigue and serving for mechanical functions as energy stores, power amplifiers, cushioning impacts and force control [19, 20].

Here we present newly discovered protein from *C*. *quadricarinatus* possessing a chitin binding domain and some similarity to the pro-resilin family of cuticular proteins. We identified and characterized the above protein, which we termed Cq-M13, and verified its transcript in a molar tooth epithelium cDNA. The temporal level of transcription of *Cq-M13* found in an NGS library constructed from molar-forming epithelia at different molt stages, perfectly matched the assembly and mineralization pattern of the molar tooth. Functionally, *in vivo* silencing of the transcript caused molt cycle delay and a recombinant version of the protein was found to bind chitin and possess a certain level of elasticity. Further, we used indentation as a nondestructive method of characterizing mechanical properties of materials [21, 22]. Indentation is routinely used for measuring mechanical properties at the submicrometer scale [23]. Indentation showed that Cq-M13 possesses elasticity properties.

## Materials and Methods

### Animals

*C*. *quadricarinatus* males were grown at Ben-Gurion University, under conditions described previously [3]. Inter-molt crayfish were endocrinologically induced to enter pre-molt through injection of ecdysone [3]. The molt stage was monitored using the molt mineralization index (MMI) [3]. For dissections, crayfish were placed on ice for 5–10 min until anesthetized.

### Purification of molar proteins

Inter-molt crayfish were endocrinologically induced as above and 14 days later, their molar teeth were dissected at late pre-molt stage. Forty molar teeth from crayfishes weighing between 60 g to 70 g were pulverized in liquid nitrogen using a mortar and pestle and proteins were extracted as previously described [24]. The molar protein extract was separated on a 2D protein gel as described previously [25]. Spots were visualized by Coomassie Blue staining and gel pieces were excised for mass spectrometry.

### Mass spectrometry

The proteins excised from the gel were digested with trypsin (Gold Trypsin, Promega) and the resultant peptides were purified on a C-18 column and dissolved in 0.1% formic acid. Nanoliquid chromatography and mass-spectrometry analysis were performed as described previously [25]. Mass spectra were acquired using an LTQ-Orbitrap XL (Thermo Fisher Scientific, San Jose, CA) and analyzed using the PEAKS Mass Spectrometry Software, including a *de novo* peptide sequencing approach. Further validation was performed by the Sequest and Mascot algorithms operated under Proteome Discoverer 2.0 (Thermo Fisher Scientific) using a NCBI database containing the newly identified Cq-M13 sequence.

### Transcript sequencing

Degenerative primers Cq-M13 dF (5'-caY tty acN Gcn yti wsi GC-3') and Cq-M13 dR (5'-CGN CGN ATR GGy GGy GGy GG-3') were designed based on the sequence of a partial fragment of Cq-M13 protein obtained by tandem MS. A corresponding cDNA fragment was amplified using this primer pair. The PCR conditions using REDTaq ReadyMix PCR Reaction Mix (Sigma-Aldrich, Rehovot, Israel) were: 94°C for 3 min, followed by 30 cycles of 94°C for 30 s, 52°C for 30 s, and 72°C for 3 min, followed by a final elongation step of 72°C for 10 min. The fragment was cloned into the pGEM-T Easy vector (Promega, Madison, WI) and sequenced. 5'- and 3'-rapid amplifications of cDNA ends were carried out with the SMART rapid amplification of cDNA ends kit (Clontech, Mountain View, CA), according to the manufacturer’s protocol to obtain the entire sequence of *Cq-M13*.

### Cq-M13 bioinformatic analysis

The deduced amino acid sequence of the gene was obtained bioinformatically by the ExPASy Translate tool [26]. Signal peptide and putative phosphorylation sites were predicted using SignalP 4.0 [27] and NetPhos 2.0 [28], respectively. Domain presence was predicted by SMART [29, 30] and Motif Scan [31] and its type-by a CuticleDB server [14]. Physico-chemical parameters of the Cq-M13 protein sequence including molecular weight and amino acid composition, were predicted using ProtParam tool [32]. Secondary structure predictions were done by PSIPRED [33].

### Phylogenetic analysis

A search for homologous sequences to Cq-M13 (www.ncbi.nlm.nih.gov) and their phylogenetic analysis among arthropod sequences was performed by the neighbor-joining method [34] using MEGA5 software [15]. Evolutionary distances were computed using the Poisson correction method [8] and are expressed as amino acid substitutions per site. All positions containing gaps and missing data were eliminated from the analysis.

### Accession number

*Cq-M13* sequence data has been deposited in the GenBank Data Bank with accession number: KM923892.

### Three-dimensional tomography

To study the development of the molar tooth during the molt cycle, mandibular samples were imaged with a SkyScan 1174 X-ray microtomograph employing a 50 keV/40 W tungsten x-ray source and a 1.3 megapixel CCD camera. Scans were performed using a pixel size of 6.5 μm, over 360 degrees with a rotation step of 0.7 degrees. The images were reconstructed using the SkyScan NRecon program, analyzed with SkyScan CTAn software; 3D pictures were generated using SkyScan CTvox. The system employs a ring-artifact-reduction and beam-hardening correction utility during reconstruction, which was engaged for all the images presented.

### *In silico* Cq-M13 transcription pattern

A reference *C*. *quadricarinatus* transcriptome was constructed from sequencing of four sets of samples: [35] RNA extracted from hypodermis, gastrolith and molar forming epithelium sequenced paired-end 100 bp by HiSeq2000 apparatus (Illumina, San Diego, CA) at the Applied Genomics Institute (Udine, Italy). (2) RNA extracted from molar and gastrolith forming epithelium from different molt stages (inter-molt, early pre-molt, late pre-molt and post-molt), barcoded and sequenced single-end 50 bp by HiSeq2000 apparatus (Illumina, San Diego, CA) at the Applied Genomics Institute (Udine, Italy). (3) RNA sequenced by 454 technology, as described by Glazer et. al. [36]. (4) RNA sequenced "single pass" by Sanger technology ("Expressed Sequence Tags") as described by Yudkovsky et al. [37]. All sequences (also called "reads") were assembled *de novo* with CLC Genomics Workbench 6.51 (CLC Bio, Aarhus, Denmark) using default parameters. The resulting contigs were submitted to a second assembly by CAP3, yielding a collection of putative transcripts regarded as the "Reference C. *quadricarinatus* transcriptome". The 50 bp sequences from molar and gastrolith samples at different molting stages as described above were aligned to the reference C. *quadricarinatus* transcriptome using STAR 2.3. For estimation of transcript expression levels, the number of sequences aligned to each transcript at each stage was counted, and further normalized to the total number of sequences in that stage. A statistical test for assessing the differences in expression among stages was carried out using DESeq [38]. The *Cq-M13* sequence was searched against the reference transcriptome using BLAST, and the transcript with the highest score and lowest e-value was regarded as the *Cq-M13* transcript at different molt stages: inter-molt (pool of animals, n = 1), early pre-molt (pool of animals, n = 1), late pre-molt (two single animals and one pool, n = 3) and post-molt (two single animals, n = 2).

### Temporal and spatial transcription

RNA was extracted from the forming epithelia of molar, cuticle, basal segment, and mandible tissues, as well as from the hepatopancreas, muscle and testis, using EZ-RNA Total RNA Isolation Kit (Biological Industries, Beit Haemek, Israel), according to the manufacturer’s protocol. First strand cDNA was generated with oligo (dT) 22 VN using expand RT (Roche, Basel, Switzerland). PCR was performed with the Cq-M13F (5'-gggcgagccaggtgactatggacc-3') and Cq-M13R (5'- ccaccctttgtttgcgaccgtcg-3') primers. 18S rRNA was evaluated as described previously [25]. *Cq-M13* was amplified by means of PCR with REDTaq ReadyMix PCR Reaction Mix (Sigma); using specific conditions: 94°C for 1 min, followed by 30 cycles of 94°C for 1 min, 60°C for 2 min, 72°C for3 min, followed by 10 min at 72°C).

### Cq-M13 silencing and real time RT-PCR

*Cq-M13* and *CqVg* (*C*. *quadricarinatus* vitellogenin) dsRNAs were synthetized as described previously [39]. In brief, single-stranded RNAs were synthesized based on ORFs of *Cq-M13* and *CqVg* with a MEGAscript T7 kit (Ambion, Inc., Austin, TX) according to the manufacturer’s instructions. Sense and antisense RNA were purified and hybridized as described previously [2]. Inter-molt males (MMI = 0; 5–10 g) were injected with ecdysone to induce molting [25], and either siRNA targeting Cq-M13—*Cq-M13* dsRNA, 5μg/g (treatment group; n = 5) or *CqVg* dsRNA [40], 5μg/g (control group; n = 4). The concentration of the ecdysone (Sigma-Aldrich) was 1 ng/μl [2], applied into the sinus of the first abdominal segment. On the eleventh day, animals were dissected to isolate the cuticle, molar-forming epithelia and muscle. RNA was extracted as described above from the muscle and the forming epithelium of the molar and cuticle from experimental and control groups. First strand cDNA was generated by reverse transcription using random hexamer primers (VersocDNA kit, Thermo Fisher Scientific, Waltham, MA). Relative quantification of *Cq-M13* transcript levels was obtained using the following primers: Cq-M13qPCR_ F (5'- ACgACTCTgATATCgAACCA-3'), Cq-M13qPCR_R (5'- TTTTTTTTTTgTgATACgTAATgA-3') and Probe TM1 6FAM-TTCCACATTTACCCTATAA+A+A+T+AT-BBQ, especially designed by TIB MOLBIOL Synthese labor (Roche, Basel, Switzerland). *Cq-18S* (accession no. AF235966), was used as the housekeeping gene and was evaluated in all the experiments by real time RT-PCR under similar conditions [25].

### Recombinant protein production

Due to the very small amounts and difficulty of extraction from tissue samples, in order to elucidate Cq-M13 functions *in vitro*, rCq-M13 was produced and purified and verified through Western blot by GenScript (GenScript Inc., Piscataway, NJ). In brief, rCq-M13 was chemically synthesized, cloned into pUC57-Kan vector and expressed in *E*. *coli* BL21 (DE3) at 37°C with 200 rpm agitation. SDS—PAGE and Western blot were used to detect protein expression.

### Chitin-binding assay

A chitin-binding assay was performed as described previously [41]. Gastrolith protein extract was obtained as described elsewhere [2]. Briefly, a solution of rCq-M13 was incubated with chitin powder followed by successive washes starting with DDW and then with 0.2 M NaCl and finally boiled for 10 min in 2% SDS, 20% (v/v) β-mercaptoethanol. The supernatant of each wash was electrophoresed on 15% SDS-PAGE and bands were visualized by silver staining. BSA served as a negative control and GAP 65 served as a positive control.

### Nanoindentation for determining mechanical properties

The full length rCq-M13 was dried on the surface of a mica slide. A total number of six independent trials were performed with rCq-M13 samples. Nano-indentations were performed on an Agilent XP nanoindenter using the dynamical contact module (DCM) head and a Berkovich tip. Indenter tip was approached to the surface at a rate of 10 nm/s using the continuous stiffness measurement (CSM) mode where a small (1 nm) oscillation was imposed on the indenter tip at 75 Hz. Surface detection was performed when the contact stiffness exceeded the nominal instrumental spring stiffness of 100 N/m. The indentation was performed to depths of 250 nm into the surface at strain rate of 0.05 s^-1, and the modulus reported was calculated using the Oliver and Pharr formula for the CSM signal, averaged over depths between 100–220 nm [23]. Elasticity moduli are presented in gigapascal (GPa).

### Construction of a cDNA library of the molar-forming epithelia

Total RNA isolation and cDNA preparation were performed following the dissection of molar- and cuticle-forming epithelium as previously described by us [42]. The cDNA was then used to prepare a subtraction library of the molar-forming epithelium with the Clontech PCR Select cDNA Subtraction Kit (BD Biosciences, San Jose, CA, following the manufacturer’s instructions, using the cDNA from molar-forming epithelium as the tester and the cDNA from cuticle-forming epithelium as the driver. After two hybridization cycles, unhybridized cDNAs, representing genes that are expressed in the molar-forming but are absent from the cuticle-forming epithelium, were amplified by two PCRs. The primary (27 cycles) and secondary (20 cycles) PCRs were performed as recommended in the Takara DNA polymerase manual and the PCR products were cloned into the pGEM-Teasy vector (Promega), and sequenced. To enhance the quality of the selected expressed sequence tags (ESTs), the obtained cDNA sequences were first stripped of low quality vector and primer sequences using Sequencher software (GeneCodes Corp.) and the clean sequences were assembled. Functional annotation of the resulting sequences of contigs and singlets was carried out by conducting a BLASTX search of each transcript against the UniRef90 database. The Blast2GO software suite [43, 44] was used to predict transcript function and assign Gene Ontology terms [45, 46]. Blast2GO parameters included BLASTX against the GenBank non-redundant (nr) database hosted by the National Center for Biotechnology Information (NCBI) (http://www.ncbi.nlm.nih.gov/), with an e-value threshold <1e^-6^, retrieving 3 blast hits for each transcript. Mapping and annotation were performed using default parameters.

### Statistical analyses

Data are expressed as means ± SE. Non-parametric tests were used as follows: Statistical analysis for relative transcript levels among different tissues and molt stages was performed using the Kruskal—Wallis rank sum test, followed by multiple pair-wise comparisons using the Wilcoxon rank sum test. *P* values <0.05 were considered statistically significant.

## Results

**Fig 1:**
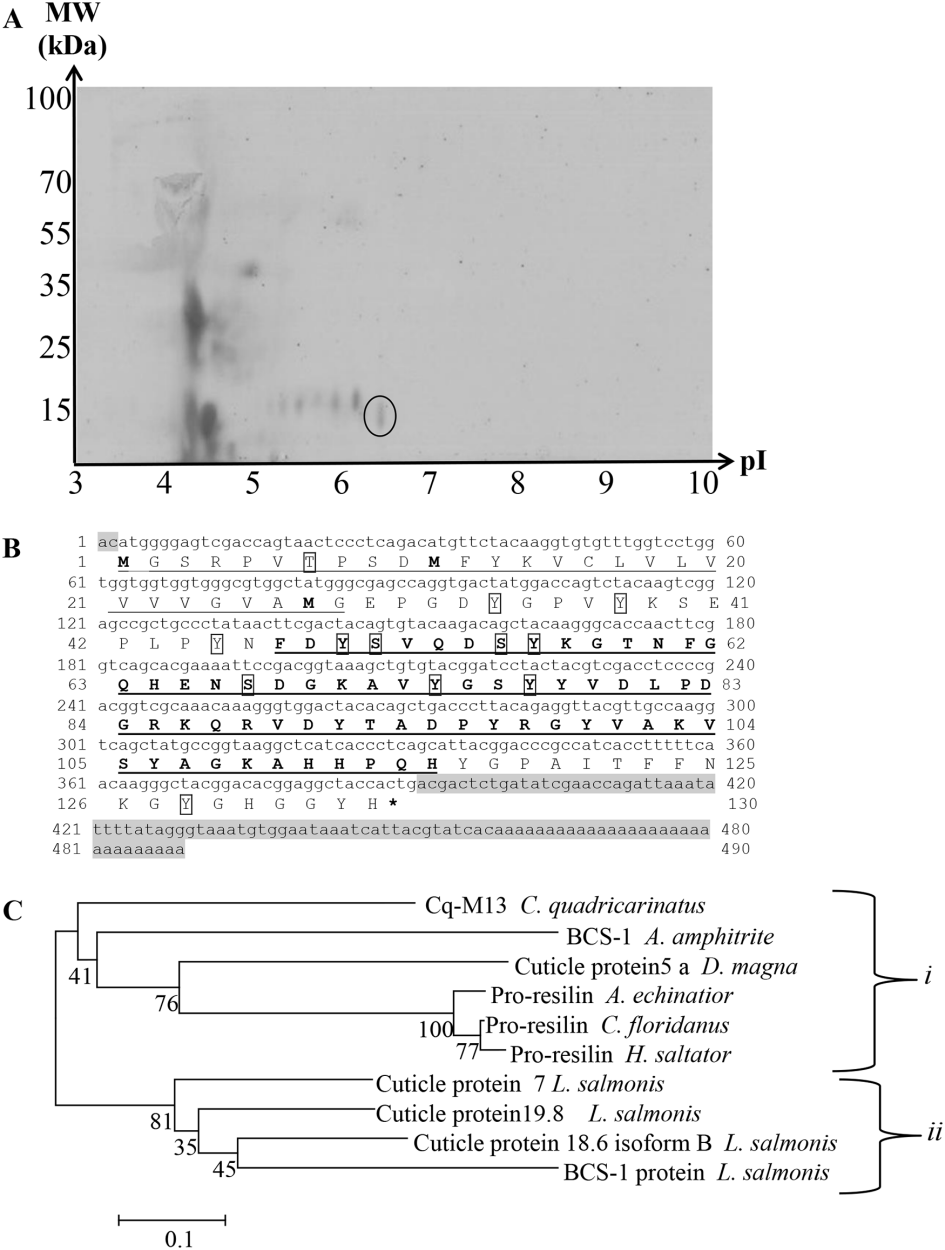
Two-Dimensional electrophoresis of *C*. *quadricarinatus* molar proteins and bioinformatic and phylogenetic analysis of Cq-M13. **A**. Proteins were separated on a linear 3–10 pH gradient and then on 12% polyacrylamide gel stained with mass compatible Coomassie blue. Cq-M13 was identified in the spot of ~14 kDa and pI ~5.9 (the spot is marked by a circle). **B**. Nucleotide sequence of Cq-M13 cDNA and deduced amino acids of its open reading frame. The 5' and 3' untranslated regions are highlighted in gray. The putative signal peptide in the N-terminus is underlined. The predicted chitin binding R-R domain is marked in bold and underlined. Predicted phosphorylation sites are boxed. The asterisk indicates a stop codon. **C**. Phylogenetic analysis of Cq-M13 in comparison to cuticular proteins from crustaceans and insects. The phylogenetic tree based on the *C quadricarinatus* Cq-M13 protein sequence and those of homologous proteins from selected invertebrates shows two distinct clades: one clade (i) comprising cuticular proteins belonging to the Resilin family and another clade (ii) comprising cuticular proteins expressed in arthrodial (non-calcified) cuticles of unknown function. The numbers shown next to the branches indicate the percentage of replicate trees in which the associated taxa clustered together in the bootstrap test. The branch lengths correspond to evolutionary distances (expressed in number of amino acid substitutions per site) used to infer the phylogenetic tree.

In screening the protein profile extracted from the molar tooth using 2D gel electrophoresis, several proteins were found. Each of the spots on the gel were excised and subjected to mass spectrometry for peptide *de novo* sequence analysis. Based on the peptide sequences obtained, degenerate primers were constructed, cDNA fragments were amplified using the molar forming epithelium RNA as template. Among the screened proteins, a protein termed Cq-M13 was identified from one 2D PAGE spot, of ~14 kDa and pI of ~5.9 (Fig 1A). Eventually its full transcript was obtained (Fig 1B).

From a cDNA subtractive library constructed from molar-forming epithelium of *C*. *quadricarinatus*, 200 DNA sequence fragments were assembled into 178 contigs and 22 singlets, together encompassing 47 putative genes. As shown in S1 Fig, 21% of the assembled sequences share no significant similarity (*E* value>0.01) to any known genes or proteins in the database; however, 39% of the assembled sequences share significant similarity with gene encoding hypothetical protein with no annotated function in the sea anemone *Nematostella vectensis*. The remaining 31% of sequences were related to a wide range of functions including protein metabolism, transferase activity, transport, structural proteins, glucose metabolism, DNA metabolism, and ion binding. *Cq-M13* comprises 9% of the assembled sequences of a cDNA subtractive library constructed from molar-forming epithelium of C. *quadricarinatus* (S1 Fig).

The 490 bp transcript of Cq-M13 includes a putative 5' UTR of 2 bp, an ORF of 390 bp encoding a deduced 130 amino acid protein, and a putative 3' UTR of 98 bp (Fig 1B). The first 28 amino acids of the predicted protein product correspond to a signal peptide, in accordance with the detection of the protein in the extracellular matrix. The predicted pI of Cq-M13 is 7 and possible phosphorylation sites are suggested on three serine residues, seven tyrosine residues and one threonine residue. Cq-M13 is predicted to be a hydrophilic protein (GRAVY = -0.571), with estimated charge of 1.3 at pH 7.00. Bioinformatics analysis also suggests the presence of a chitin-binding R-R domain spanning residues 31–102. This domain is assigned to the RR-2 family, members of which have been localized to hard cuticles [47]. Following the assembly of its sequence and comparing to the NCBI protein database, the putative Cq-M13 was found to be similar to cuticular proteins found in crustaceans and insects. Evolutionarily, these proteins were found to be assigned into two clades (Fig 1C). Clade i include Cq-M13 and cuticular proteins belonging to a pro-resilin protein family, while clade ii includes cuticular proteins expressed in arthropod cuticles with unknown function. From the evolutionary perspective, Cq-M13 is closer to pro-resilin proteins from arthropod cuticles [48].

**Fig 2:**
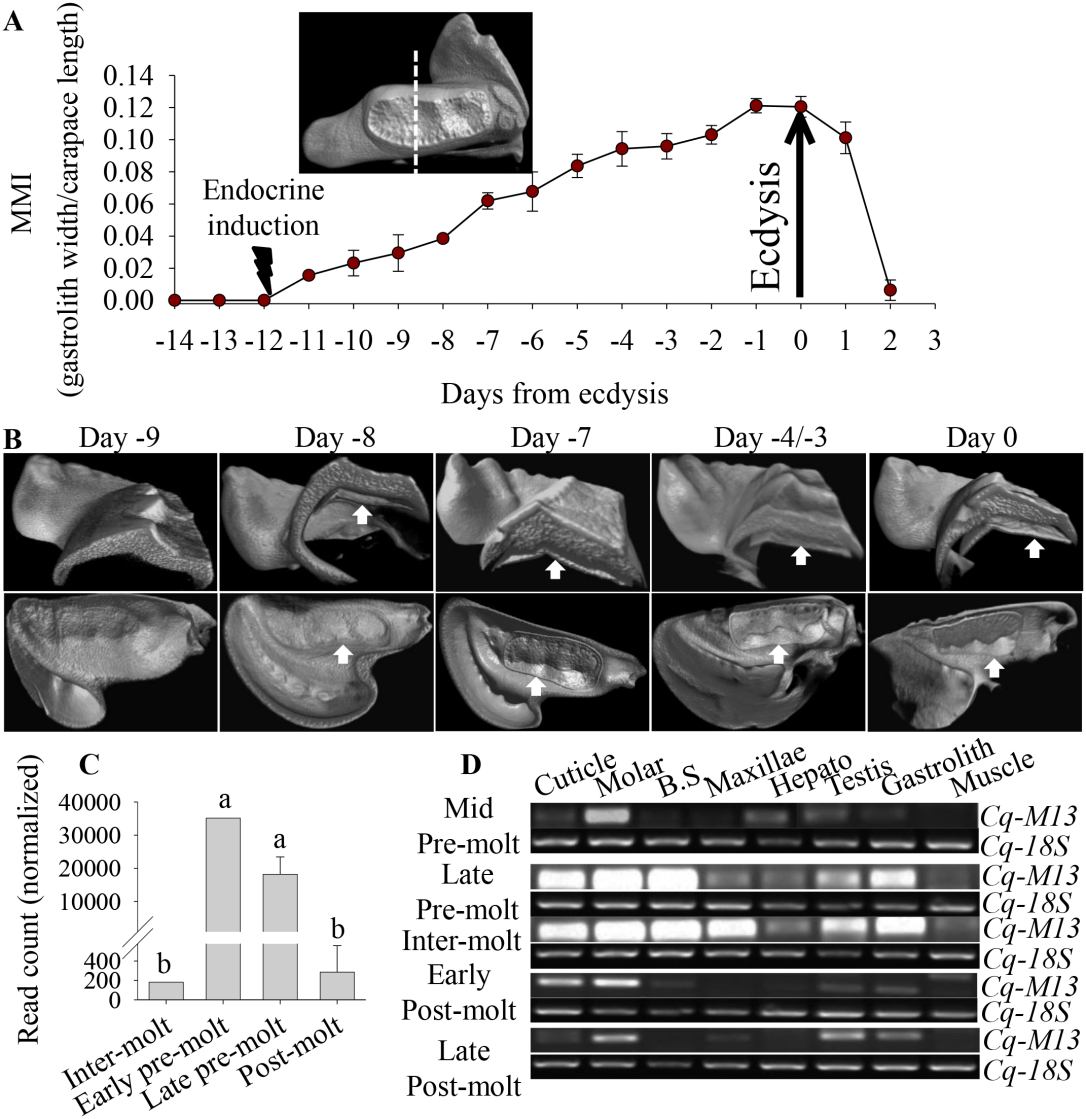
Development of a new molar tooth during an induced molt cycle in an ecdysone-injected male *C*. *quadricarinatus* and spatial and temporal expression patterns of the *Cq-M13* transcript *in vitro* and *in silico*. **A**. Changes in molt mineralization index (MMI) during the induced molt cycle following repetitive injections of ecdysone. The x axis is normalized to days from ecdysis. A dashed line on an isolated mandible represents a cut through which a transverse plane is visible (A-top). **B**. Visualization of the new molar tooth assembly process in the crayfish mandible on days -9, -8, -7, -4, -3 and 0, following ecdysone injection by *ex vivo* micro-computed tomography. The top and bottom series of images show a view of the transverse and posterior planes of the mandibles correspondingly. White arrows point to the newly formed molar tooth. **C**. *In silico* transcriptomic analysis of *Cq-M13* read counts from four different molt stages. Different letters above columns represent statistically significant differences (p < 0.05 ± SE). **D**. Agarose gels showing RT-PCR products demonstrating spatial and temporal expression patterns of the *Cq-M13* transcript in cuticle-, molar-, basal segment (B.S.)-, maxillae- and gastrolith-forming tissues, as well as in hepatopancreas (Hepato) and testis.

The molt cycle of *C*. *quadricarinatus* was induced and monitored using the MMI index (Fig 2A, modified) [49]. The *ex vivo* micro-computed tomography (Fig 2B) did not show new molar formation on day -9 (in relation to ecdysis on day 0), but from day -8 a clear assembly of a new molar could be detected. As new molar tooth formation progressed through the molt cycle, its separation from the old molar tooth became apparent starting from day -4, whereas on day 0 (on ecdysis) a fully formed and mineralized new molar tooth is visible beneath the old one (Fig 2B). As we found that Cq-M13 possesses similarity to exoskeletal proteins from mineralized crustacean cuticles (Fig 1C), and since the molar tooth is built during pre-molt (Fig 2B), a differential expression analysis of the *Cq-M13* transcript was performed. RNA sequencing of hypodermis, gastrolith and molar forming tissue by Illumina (560 million reads), and additional 454 and Sanger sequencing (287,900 reads) was followed by *de novo* assembly, yielding 56,490 contigs. Based on the above database the differential expression analysis of the *Cq-M13* transcript is significant increased (p <0.05) in a normalized read count of early- and late- pre- molt compared to the post- and inter- molt stages at the molar forming epithelium samples (Fig 2C). In addition, the specific transcription of *Cq-M13* was tested at different molting stages in a variety of target tissues by RT-PCR, revealing that *Cq-M13* is transcribed throughout all molt stages in exoskeletal element forming tissues, such as the cuticle, molar, basal segment, maxillae and gastrolith, and was even detected in non-exoskeletal tissues such as the hepatopancreas and testis (Fig 2D). Thus Cq-M13 transcription is perfectly matched with the RNA sequencing results (Fig 2C) and mineralization of the molar tooth (Fig 2B).

**Fig 3:**
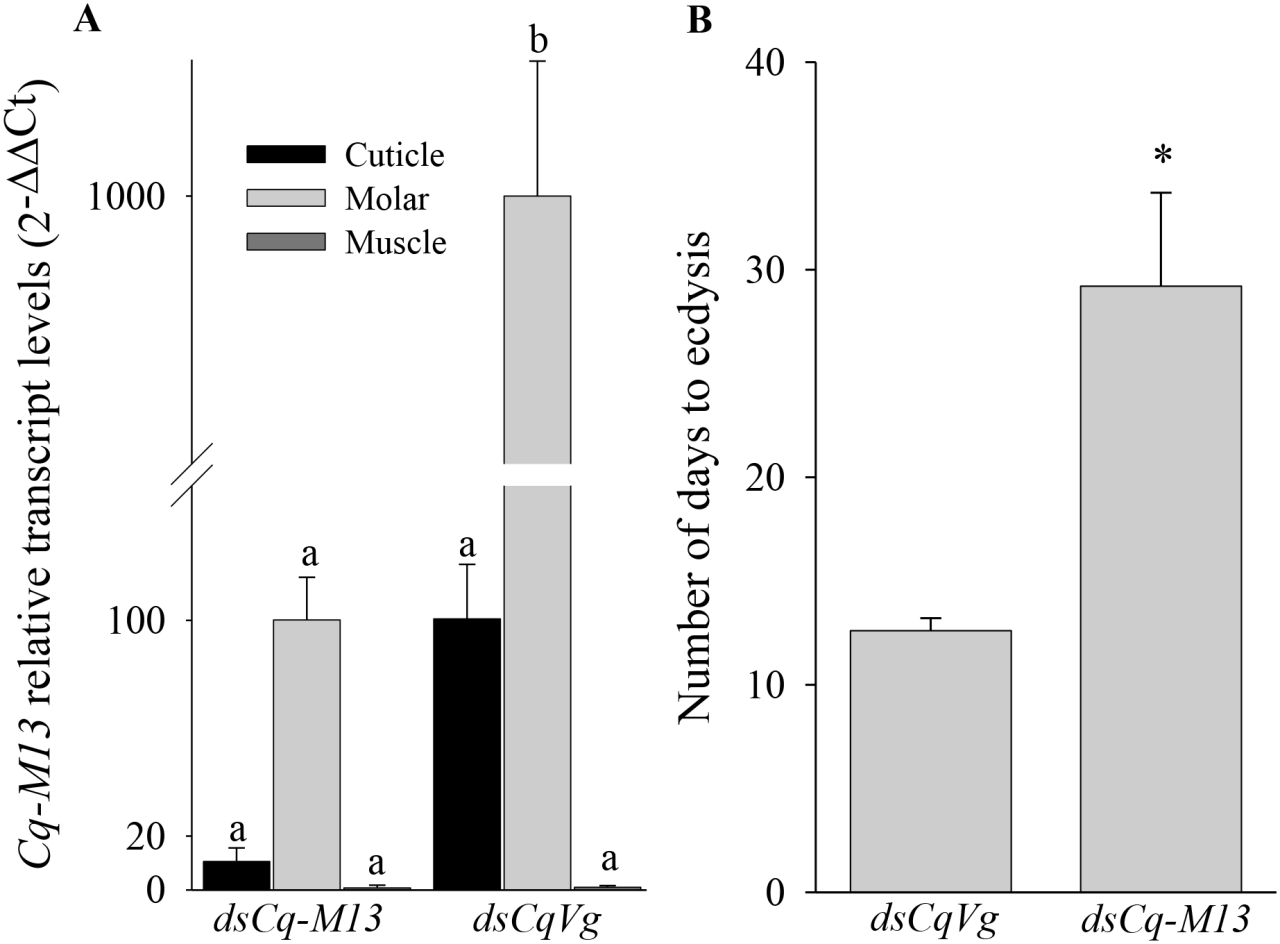
*Cq-M13* silencing effects. **A**. Levels of *Cq-M13* transcripts following *in vivo* dsRNA injections in male crayfish, as assessed by real-time RT-PCR following short-term silencing. Animals were injected with ecdysone and either ds*Cq-M13* or ds*CqVg*. Different letters represent significant differences and error bars represent standard error (p < 0.05 ± SE). **B**. Molt cycle progress elongation of *Cq-M13*-silenced *C*. *quadricarinatus* males. The experimental group was injected with *Cq-M13* dsRNA and the control group was injected with *CqVg* dsRNA. Both groups were injected with ecdysone to induce their molt cycle. Asterisk represents a significant difference (p < 0.05± SE).

A study of *Cq-M13* function *in vivo* through an RNAi loss of function approach was attempted by *Cq-M13* dsRNA administration. First, to rule out non-specific silencing of other non- *Cq-M13* transcripts in the crayfish, the *Cq-M13* dsRNA sequence was compared with all known transcripts listed in *C*. *quadricarinatus* databases [37, 50]; no similar sequences were detected. Relative *Cq-M13* transcript levels were quantified in crayfish males by real-time RT-PCR following silencing for 10 days during the molt cycle (Fig 3A). Animals were injected with either *ds Cq-M13* (n = 5) or *dsCqVg* (n = 4) and ecdysone. Transcript levels were found to be statistically different between the two groups (Kruskal-Wallis chi-squared = 22.3899, df = 5, p-value < 0.001). Wilcoxon rank sum test showed a significantly lower transcription level of *Cq-M13* in the experimental group in the molar-forming (p<0.05) epithelium compared to *Cq-M13* transcription in control molar-forming epithelium. Moreover, silencing the expression of *Cq-M13* was found to prolong molt cycle by almost two fold (Fig 3B).

**Fig 4:**
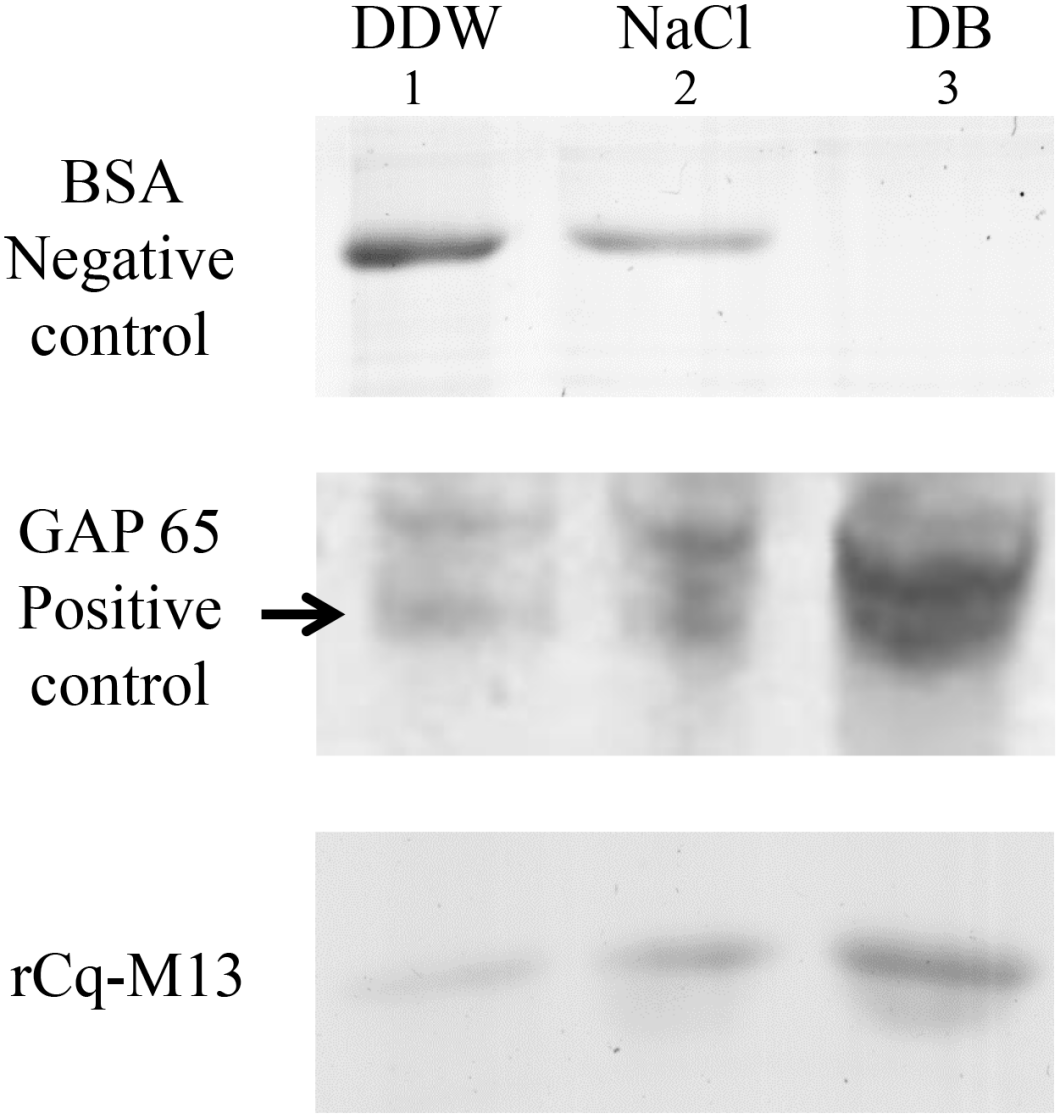
Chitin-binding ability of rCq-M13. Following incubation with chitin powder, equal amounts of Negative control—BSA (top), Positive control—GAP 65 (middle, using gastrolith protein extract with known chitin binding abilities [2], and rCq-M13 (bottom) underwent three consecutive washes prior to SDS-PAGE. Lane 1 represent the first wash with DDW, lane 2 represent the second wash with NaCl (0.2 M) and lane 3 represent the third wash with denaturation buffer (DB, containing SDS and 2-mercaptoethanol).

Recombinant Cq-M13 (rCq-M13) was used to study chitin binding properties of the protein *in vitro*. In agreement with the bioinformatics-based prediction of Cq-M13 containing a chitin-binding domain, rCq-M13 was shown to specifically bind chitin. Unbound rCq-M13, bovine serum albumin [51] (which served as a negative control protein, lacking a chitin-binding domain) and GAP 65 (which served as a positive control protein, lacking a chitin-binding domain) were recovered in the supernatat after consecutive washes of a chitin-containing solution with DDW (Fig 4) and were also detected following further washings with weak salt solution (Fig 4A). To identify the proteins that were more strongly bound to chitin, harsher wash conditions were employed. The majority of rCq-M13 was recovered only after boiling the washed chitin in denaturation buffer (DB) containing SDS and 2-mercaptoethanol) (Fig 4, lane 3).

To characterize mechanical properties of the protein at the submicrometer scale we measured the elastic modulus, which reflects the resistance to being deformed when a force is applied on it [52]. We also measured the hardness, which is the mean pressure built up under the indenter at its maximal depth [52]. The average values of elastic modulus and the hardness from full length recombinant Cq-M13 are 6.82±2.52 GPa and 0.32±0.1 GPa correspondingly, showing that the protein possesses some degree of elasticity.

## Discussion

In this study, first we followed the dynamics of the new molar calcification during *C*. *quadricarinatus* molt cycle. The rapid accumulation of mineral in the molar during pre-molt coincides with a simultaneous increase in gastrolith mineral density and a decrease in cuticle density prior to ecdysis previously described by us [3]. Thus molar calcification occurs at the pre-molt stage, similar only to the gastrolith and unlike the rest of the new exoskeleton which is not mineralized at this point [3]. The importance of proteins in biomineralization is unequivocal [53]. The present study marks the initiation of a long journey towards a complete picture of molar tooth proteins presence and function. The description of temporal process of mineralization might enable the study of molar transcripts and their proteins with relationship to the temporal changes in molar calcification, such as above mentioned putative transcripts identified from a cDNA subtractive library. An interesting candidate for future investigation might be the one that constitutes 39% of the SSH library and shares significant similarity with a gene encoding hypothetical protein from the sea anemone, *Nematostella vectensis*, with no annotated function.

A novel transcript termed *Cq-M13* and its protein product were identified from the crayfish molar tooth and its characterization suggests its possible involvement in the molar tooth assembly. Based on the results obtained by Real-Time PCR we would assume that the magnitude of transcription of *Cq-M13* is lower than in the mouthparts. However, some genes have been reported to possess a dual function [54–57]. Based on the example of the *ovo* gene that is required for cuticle formation [58] and oogenesis in flies [59, 60], however this study was performed on male crayfish while the suggestion that *Cq-M13* might be involved in oogenesis has to be further studied in females. Nonetheless, the transcription of *Cq-M13* during the pre-molt stage coincides with molar tooth assembly during this particular molt stage (11) and presumably indicates its involvement in exoskeleton assembly.

Furthermore, *in vivo* silencing of *Cq-M13* delayed molt. Gene silencing studies assessing the functionality of novel crustacean genes have been previously performed [61–63]. We have previously shown that silencing of particular transcripts in *C*. *quadricarinatus* affected the formation of the extracellular matrix and the calcification process [2, 25], as well as the duration of molt cycles [64]. However, it should be noted that although a complete sequence search was done prior to the silencing study, the lack of a sequenced genome for *C*. *quadricarinatus* makes it impossible to completely rule out potential effects of non-specific small interfering RNAs derived from the *Cq-M13* sequence acting within other target transcripts.

Cq-M13 is predicted to contain the RR-2-type chitin-binding motif. Cq-M13 was extracted from a hard cuticle, i.e. the molar tooth, correspondingly with the location of most proteins containing this motif, which are found in hard cuticular elements [47]. Since hard cuticles are often exposed to strong forces, it can be speculated that RR-2 containing proteins are strongly attached to chitin to prevent detaching from it [47, 65]. In agreement with the bioinformatics-based prediction, rCq-M13 was found to possess chitin-binding ability *in vitro*, thus suggesting its possible direct association with the extracellular chitinous matrix. The RR-2 consensus sequence was also found in the pro-resilin protein family [66, 67]. The term resilin refers to mature, fully crosslinked material, whereas the not-yet crosslinked protein is termed pro-resilin [48]. Resilin is a type of elastic protein and as such is characterized by being able to undergo deformation, and to return to its original state after the stress is removed [68]. According to a phylogenetic analysis Cq-M13 was found to share significant similarity to pro-resilin proteins from arthropod cuticles. In accordance with previous investigations regarding features attributed to the pro-resilin family [69, 70], Cq-M13 contains an N-terminal signal sequence, indicating that it is a secreted protein; it is dominated by high tyrosine, glycine and proline content and possesses an RR-2 consensus sequence. In accordance with these features, the nanoindentation measurements demonstrated that rCq-M13 exhibits some elasticity and hardness. rCq-M13 is much less elastic than resilin, which has an elasticity modulus of 0.002 GPa [71] and collagen which has an elasticity modulus of 1.2 GPa [72] and more elastic than dragline silk, which has an elasticity modulus of 10 GPa [73]. These data suggest rCq-M13 might serve as a flexible material that is employed together with the chitinous cuticle in a composite structure for efficient energy storage. However, it will be necessary to further investigate whether or not Cq-M13 is crosslinked by dityrosine residues to form an insoluble elastic material, before we can decide whether it is similar to pro-resilin in function [66] with respect to its contribution to the elasticity of exoskeletal elements.

## Supporting Information

S1 Fig Gene Ontology (GO) terms assigned to the 200 ESTs (47 putative genes) from a *C quadricarinatus* molar-forming epithelium cDNA subtractive library. More than 21% of the sequences had no significant similarity (*E*-value > 0.01) to any Uniprot protein. 39% were similar to hypothetical protein of sea anemone *N*. *vectensis*, 9% were similar to Cq-M13, and the rest could be related to proteins associated with the indicated GO categories.
(TIF)

## References

[1] Bentov S, Zaslansky P, Al-Sawalmih A, Masic A, Fratzl P, Sagi A, et al Enamel-like apatite crown covering amorphous mineral in a crayfish mandible. Nat Commun. 2012;3:839 10.1038/ncomms1839 22588301 PMC3382302

[2] Shechter A, Glazer L, Chaled S, Mor E, Weil S, Berman A, et al A gastrolith protein serving a dual role in the formation of extracellular matrix containing an amorphous mineral. Proc Natl Acad Sci U S A. 2008;105(20):7129–34. 10.1073/pnas.0800193105 18480260 PMC2438216

[3] Shechter A, Berman A, Singer A, Freiman A, Grinstein M, Erez J, et al Reciprocal changes in calcification of the gastrolith and cuticle during the molt cycle of the red claw crayfish *Cherax quadricarinatus* . Biol Bull. 2008;214(2):122–34. 18400994 10.2307/25066669

[4] George A, Veis A. Phosphorylated Proteins and Control over Apatite Nucleation, Crystal Growth, and Inhibition. Chem Rev. 2008;108(11):4670–93. 10.1021/cr0782729 18831570 PMC2748976

[5] Glazer L, Sagi A. On the involvement of proteins in the assembly of the crayfish gastrolith extracellular matrix. Invertebr Reprod Dev. 2012;56(1):57–65.

[6] Luquet G. Biomineralizations: insights and prospects from crustaceans. Zookeys. 2012(176):103–21. 10.3897/zookeys.176.2318 22536102 PMC3335408

[7] Nagasawa H. Structure and Function of Matrix Proteins and Peptides in the Biomineral Formation in Crustaceans In: Müller WEG, editor. Molecular Biomineralization. Progress in Molecular and Subcellular Biology. 52: Springer Berlin Heidelberg; 2011 p. 315–29. 21877271 10.1007/978-3-642-21230-7_11

[8] Lu Y, Ye L, Yu S, Zhang SB, Xie Y, McKee MD, et al Rescue of odontogenesis in Dmp1-deficient mice by targeted re-expression of DMP1 reveals roles for DMP1 in early odontogenesis and dentin apposition in vivo. Dev Biol. 2007;303(1):191–201. 17196192 10.1016/j.ydbio.2006.11.001 PMC2059935

[9] Beniash E. Biominerals-hierarchical nanocomposites: the example of bone. Wiley Interdiscip Rev Nanomed Nanobiotechnol. 2011;3(1):47–69. 10.1002/wnan.105 20827739 PMC3012754

[10] Deshpande AS, Fang PA, Simmer JP, Margolis HC, Beniash E. Amelogenin-collagen interactions regulate calcium phosphate mineralization *in vitro* . J Biol Chem. 2010;285:19277–87. 10.1074/jbc.M109.079939 20404336 PMC2885206

[11] Fang PA, Conway JF, Margolis HC, Simmer JP, Beniash E. Hierarchical self-assembly of amelogenin and the regulation of biomineralization at the nanoscale. Proc Natl Acad Sci U S A. 2011;108(34):14097–102. 10.1073/pnas.1106228108 21825148 PMC3161614

[12] Magkrioti CK, Spyropoulos IC, Iconomidou VA, Willis JH, Hamodrakas SJ. cuticleDB: a relational database of Arthropod cuticular proteins. BMC Bioinform. 2004;5:138 15453918 10.1186/1471-2105-5-138 PMC522807

[13] Tellam RL, Wijffels G, Willadsen P. Peritrophic matrix proteins. Insect Biochem Mol Biol. 1999;29(2):87–101. 10196732 10.1016/s0965-1748(98)00123-4

[14] Karouzou MV, Spyropoulos Y, Iconomidou VA, Cornman RS, Hamodrakas SJ, Willis JH. *Drosophila* cuticular proteins with the R&R Consensus: Annotation and classification with a new tool for discriminating RR-1 and RR-2 sequences. Insect Biochem Mol Biol. 2007;37(8):754–60. 17628275 10.1016/j.ibmb.2007.03.007

[15] Andersen SO. Studies on proteins in post-ecdysial nymphal cuticle of locust, Locusta migratoria, and cockroach, Blaberus craniifer. Insect Biochem Mol Biol. 2000;30(7):569–77. 10844249 10.1016/s0965-1748(00)00029-1

[16] Rebers JE, Riddiford LM. Structure and expression of a Manduca sexta larval cuticle gene homologous to Drosophila cuticle genes. J Mol Biol. 1988;203(2):411–23. 2462055 10.1016/0022-2836(88)90009-5

[17] Cornman RS. Molecular evolution of Drosophila cuticular protein genes. PLoS ONE. 2009;4(12):e8345 10.1371/journal.pone.0008345 20019874 PMC2793513

[18] Tatham AS, Shewry PR. Elastomeric proteins: biological roles, structures and mechanisms. Trends Biochem Sci. 2000;25(11):567–71. 11084370 10.1016/s0968-0004(00)01670-4

[19] Andersen SO. Structure and function of resilin In: Shewry PR, Tatham AS, Bailey AJ, editors. Elastomeric proteins: Structures, Biomechanical Properties and Biological Roles. Cambridge: Cambridge University Press; 2003 p. 259–78.

[20] Alexander RM. Functions of elastomeric proteins in animals In: Shewry PR, Tatham AS, Bailey AJ, editors. Elastomeric Proteins: Structures, Biomechanical Properties and Biological Roles. Cambridge: Cambridge University Press; 2003 p. 1–13.

[21] Gilman JJ. Hardness—a strength microprobe The Science of Hardness Testing and Its Research Applications, ASM, Metals Park, Ohio: American Society for Metals; 1973 p. 51–74.

[22] Tabor D. Indentation hardness and its measurement: some cautionary comments. Philadelphia, PA: American Society for Testing and Materials; 1986.

[23] Oliver WC, Pharr GM. An improved technique for determining hardness and elastic modulus using load and displacement sensing indentation experiments. J Mater Res. 1992;7(06):1564–83.

[24] O’Brien JJ, KumariI SS, Skinner DM. Proteins of Crustacean Exoskeletons: I. Similarities and Differences among Proteins of the Four Exoskeletal Layers of Four Brachyurans. Biol Bull. 1991;181:427–41. 29304681 10.2307/1542363

[25] Glazer L, Shechter A, Tom M, Yudkovski Y, Weil S, Aflalo ED, et al A protein involved in the assembly of an extracellular calcium storage matrix. J Biol Chem. 2010;285(17):12831–9. 10.1074/jbc.M109.071068 20150428 PMC2857067

[26] Young MF, Kerr JM, Ibaraki K, Heegaard AM, Robey PG. Structure, expression, and regulation of the major noncollagenous matrix proteins of bone. Clin Orthop. 1992;281:275–94. 1499220

[27] Petersen TN, Brunak S, von Heijne G, Nielsen H. SignalP 4.0: discriminating signal peptides from transmembrane regions. Nat Meth. 2011;8(10):785–6. 10.1038/nmeth.1701 21959131

[28] He G, Ramachandran A, Dahl T, George S, Schultz D, Cookson D, et al Phosphorylation of phosphophoryn is crucial for its function as a mediator of biomineralization. J Biol Chem. 2005;280(39):33109–14. 16046405 10.1074/jbc.M500159200

[29] Suderman RJ, Dittmer NT, Kanost MR, Kramer KJ. Model reactions for insect cuticle sclerotization: cross-linking of recombinant cuticular proteins upon their laccase-catalyzed oxidative conjugation with catechols. Insect Biochem Mol Biol. 2006;36(4):353–65. 16551549 10.1016/j.ibmb.2006.01.012

[30] Schultz J, Milpetz F, Bork P, Ponting CP. SMART, a simple modular architecture research tool: Identification of signaling domains. Proc Natl Acad Sci U S A. 1998;95(11):5857–64. 9600884 10.1073/pnas.95.11.5857 PMC34487

[31] Suzuki M, Sugisaka-Nobayashi A, Kogure T, Nagasawa H. Structural and Functional Analyses of a Strong Chitin-Binding Protein-1 (SCBP-1) from the Exoskeleton of the Crayfish Procambarus clarkii. Biosci Biotechnol Biochem. 2013;77(2):361–8. 23391933 10.1271/bbb.120787

[32] Fujisawa R, Tamura M. Acidic bone matrix proteins and their roles in calcification. Front Biosci. 2012;17:1891–903. 22201843 10.2741/4026

[33] Futahashi R, Fujiwara H. Identification of stage-specific larval camouflage associated genes in the swallowtail butterfly, Papilio xuthus. Dev Genes Evol. 2008;218(9):491–504. 10.1007/s00427-008-0243-y 18712529

[34] Saitou N, Nei M. The neighbor-joining method: a new method for reconstructing phylogenetic trees. Mol Biol Evol. 1987;4(4):406–25. 3447015 10.1093/oxfordjournals.molbev.a040454

[35] Sievers F, Wilm A, Dineen D, Gibson T, Karplus K, Li W, et al Fast, scalable generation of high-quality protein multiple sequence alignments using Clustal Omega. 10.1038/msb.2011.75 21988835 PMC3261699

[36] Glazer L, Tom M, Weil S, Roth Z, Khalaila I, Mittelman B, et al Hemocyanin with phenoloxidase activity in the chitin matrix of the crayfish gastrolith. J Exp Biol. 2013;216(10):1898–904. 23393281 10.1242/jeb.080945

[37] Yudkovski Y, Glazer L, Shechter A, Reinhardt R, Chalifa-Caspi V, Sagi A, et al Multi-transcript expression patterns in the gastrolith disk and the hypodermis of the crayfish *Cherax quadricarinatus* at premolt. Comp Biochem Physiol, Part D: Genom Proteom. 2010;5(2):171–7. 10.1016/j.cbd.2010.03.010 20452841

[38] Anders S, Huber W. Differential expression analysis for sequence count data. Genome Biol. 2010;11(10):R(106). 10.1186/gb-2010-11-10-r106 PMC3218662 20979621

[39] Rosen O, Manor R, Weil S, Gafni O, Linial A, Aflalo ED, et al A Sexual Shift Induced by Silencing of a Single Insulin-Like Gene in Crayfish: Ovarian Upregulation and Testicular Degeneration. PLoS ONE. 2010;5(12):e15281 10.1371/journal.pone.0015281 21151555 PMC3000327

[40] Shechter A, Aflalo ED, Davis C, Sagi A. Expression of the reproductive female-specific vitellogenin gene in endocrinologically induced male and intersex *Cherax quadricarinatus* crayfish. Biol Reprod. 2005;73(1):72–9. 15744019 10.1095/biolreprod.104.038554

[41] Inoue H, Ozaki N, Nagasawa H. Purification and structural determination of a phosphorylated peptide with anti-calcification and chitin-binding activities in the exoskeleton of the crayfish, *Procambarus clarkii* . Biosci Biotechnol Biochem. 2001;65(8):1840–8. 11577725 10.1271/bbb.65.1840

[42] Manor R, Weil S, Oren S, Glazer L, Aflalo ED, Ventura T, et al Insulin and gender: an insulin-like gene expressed exclusively in the androgenic gland of the male crayfish. Gen Comp Endocrinol. 2007;150(2):326–36. 17094989 10.1016/j.ygcen.2006.09.006

[43] Götz S, Garcia-Gomez JM, Terol J, Williams TD, Nagaraj SH, Nueda MJ, et al High-throughput functional annotation and data mining with the Blast2GO suite. Nucleic Acids Res. 2008;36(10):3420–35. 10.1093/nar/gkn176 18445632 PMC2425479

[44] Conesa A, Gotz S, Garcia-Gomez JM, Terol J, Talon M, Robles M. Blast2GO: a universal tool for annotation, visualization and analysis in functional genomics research. Bioinformatics. 2005;21(18):3674–6. 16081474 10.1093/bioinformatics/bti610

[45] Ashburner M, Ball CA, Blake JA, Botstein D, Butler H, Cherry JM, et al Gene ontology: tool for the unification of biology. The Gene Ontology Consortium. Nat Genet. 2000;25(1):25–9. 10802651 10.1038/75556 PMC3037419

[46] Consortium TGO. The Gene Ontology project in 2008. Nucleic Acids Res. 2008;36(Database issue):D440–4. 17984083 10.1093/nar/gkm883 PMC2238979

[47] Iconomidou VA, Willis JH, Hamodrakas SJ. Unique features of the structural model of 'hard' cuticle proteins: implications for chitin-protein interactions and cross-linking in cuticle. Insect Biochem Mol Biol. 2005;35(6):553–60. 15857761 10.1016/j.ibmb.2005.01.017

[48] Andersen SO, Weis-Fogh T. Resilin. A rubberlike protein in arthropod cuticle. Adv Insect Physiol. 1964;2:1–65.

[49] Shechter A, Tom M, Yudkovski Y, Weil S, Chang SA, Chang ES, et al Search for hepatopancreatic ecdysteroid-responsive genes during the crayfish molt cycle: from a single gene to multigenicity. J Exp Biol. 2007;210(Pt 20):3525–37. 17921154 10.1242/jeb.006791

[50] Yudkovski Y, Shechter A, Chalifa-Caspi V, Auslander M, Ophir R, Dauphin-Villemant C, et al Hepatopancreatic multi-transcript expression patterns in the crayfish *Cherax quadricarinatus* during the moult cycle. Insect Mol Biol. 2007;16(6):661–74. 18092996 10.1111/j.1365-2583.2007.00762.x

[51] Shimizu S, Sabsay B, Veis A, Ostrow JD, Rege RV, Dawes LG. Isolation of an acidic protein from cholesterol gallstones, which inhibits the precipitation of calcium carbonate in vitro. J Clin Invest. 1989;84(6):1990–6. 2592569 10.1172/JCI114389 PMC304082

[52] Oliver WC, Pharr GM. Measurement of hardness and elastic modulus by instrumented indentation: Advances in understanding and refinements to methodology. J Mater Res. 2004;19(01):3–20.

[53] Addadi L, Weiner S. Biomineralization: mineral formation by organisms. Phys Scr. 2014;89(9):098003 24655281

[54] Dai X, Schonbaum C, Degenstein L, Bai W, Mahowald A, Fuchs E. The ovo gene required for cuticle formation and oogenesis in flies is involved in hair formation and spermatogenesis in mice. Genes Dev. 1998;12(21):3452–63. 9808631 10.1101/gad.12.21.3452 PMC317232

[55] Metodiev MD, Spåhr H, Polosa PL, Meharg C, Becker C, Altmueller J, et al NSUN4 is a dual function mitochondrial protein required for both methylation of 12S rRNA and coordination of mitoribosomal assembly. PLoS Genet. 2014;10(2):e1004110 10.1371/journal.pgen.1004110 24516400 PMC3916286

[56] Lisowsky T. Dual function of a new nuclear gene for oxidative phosphorylation and vegetative growth in yeast. Molecular and General Genetics MGG. 1992;232(1):58–64. 1552903 10.1007/BF00299137

[57] Wu H, D'Alessio AC, Ito S, Wang Z, Cui K, Zhao K, et al Genome-wide analysis of 5-hydroxymethylcytosine distribution reveals its dual function in transcriptional regulation in mouse embryonic stem cells. Genes Dev. 2011;25(7):679–84. 10.1101/gad.2036011 21460036 PMC3070931

[58] Mével-Ninio M, Terracol R, Salles C, Vincent A, Payre F. ovo, a Drosophila gene required for ovarian development, is specifically expressed in the germline and shares most of its coding sequences with shavenbaby, a gene involved in embryo patterning. Mech Dev. 1995;49(1):83–95. 7748792 10.1016/0925-4773(94)00305-7

[59] Garfinkel MD, Wang J, Liang Y, Mahowald AP. Multiple products from the shavenbaby-ovo region of Drosophila melanogaster: Relationship to genetic complexity. Mol Cell Biol. 1994;14:6809–18. 7935398 10.1128/mcb.14.10.6809-6818.1994 PMC359211

[60] Mevel-Ninio M, Terracol R, Kafatos FC. The ovo gene of Drosophila encodes a zinc finger protein required for female germ line development. The EMBO journal. 1991;10(8):2259 1712294 10.1002/j.1460-2075.1991.tb07762.x PMC452916

[61] Ventura T, Manor R, Aflalo ED, Weil S, Rosen O, Sagi A. Timing sexual differentiation: full functional sex reversal achieved through silencing of a single insulin-like gene in the prawn, *Macrobrachium rosenbergii* . Biol Reprod. 2012;86(3):90, 1–6. 10.1095/biolreprod.111.097261 22133694

[62] Ventura T, Manor R, Aflalo ED, Weil S, Raviv S, Glazer L, et al Temporal silencing of an androgenic gland-specific insulin-like gene affecting phenotypical gender differences and spermatogenesis. Endocrinology. 2009;150(3):1278–86. 10.1210/en.2008-0906 18988670

[63] Rosen O, Manor R, Weil S, Gafni O, Linial A, Aflalo ED, et al A sexual shift induced by silencing of a single insulin-like gene in crayfish: ovarian upregulation and testicular degeneration. PLoS ONE. 2010;5(12):e15281 10.1371/journal.pone.0015281 21151555 PMC3000327

[64] Pamuru RR, Rosen O, Manor R, Chung JS, Zmora N, Glazer L, et al Stimulation of molt by RNA interference of the molt-inhibiting hormone in the crayfish Cherax quadricarinatus. Gen Comp Endocrinol. 2012;178(2):227–36. 10.1016/j.ygcen.2012.05.007 22664421

[65] Andersen SO. Are structural proteins in insect cuticles dominated by intrinsically disordered regions? Insect Biochem Mol Biol. 2011;41(8):620–7. 10.1016/j.ibmb.2011.03.015 21477652

[66] Andersen SO. Studies on resilin-like gene products in insects. Insect Biochem Mol Biol. 2010;40(7):541–51. 10.1016/j.ibmb.2010.05.002 20457254

[67] Neville AC. Growth and deposition of resilin and chitin in locust rubber-like cuticle. J Insect Physiol. 1963;9(3):265–78.

[68] Gosline J, Lillie M, Carrington E, Guerette P, Ortlepp C, Savage K. Elastic proteins: biological roles and mechanical properties. Philos Trans R Soc Lond B Biol Sci. 2002;357(1418):121–32. 11911769 10.1098/rstb.2001.1022 PMC1692928

[69] Elvin CM, Carr AG, Huson MG, Maxwell JM, Pearson RD, Vuocolo T, et al Synthesis and properties of crosslinked recombinant pro-resilin. Nature. 2005;437(7051):999–1002. 16222249 10.1038/nature04085

[70] Qin G, Lapidot S, Numata K, Hu X, Meirovitch S, Dekel M, et al Expression, Cross-Linking, and Characterization of Recombinant Chitin Binding Resilin. Biomacromolecules. 2009 10(12):3227–34. 10.1021/bm900735g 19928816

[71] Weis-Fogh T. Molecular interpretation of the elasticity of resilin, a rubber-like protein. J Mol Biol. 1961;3(5):648–67.

[72] Pollock CM, Shadwick RE. Relationship between body mass and biomechanical properties of limb tendons in adult mammals. Am J Physiol. 1994;266:R1016–R. 8160850 10.1152/ajpregu.1994.266.3.R1016

[73] Denny M. The physical properties of spider's silk and their role in the design of orb-webs. J Exp Biol. 1976;65(2):483–506.

