# Supplementary figures and images for: A Novel Chitin Binding Crayfish Molar Tooth Protein with Elasticity Properties
Source: PLoS One. 2015 May 26;10(5):e0127871. doi: 10.1371/journal.pone.0127871 (PMC4444123; doi:10.1371/journal.pone.0127871)

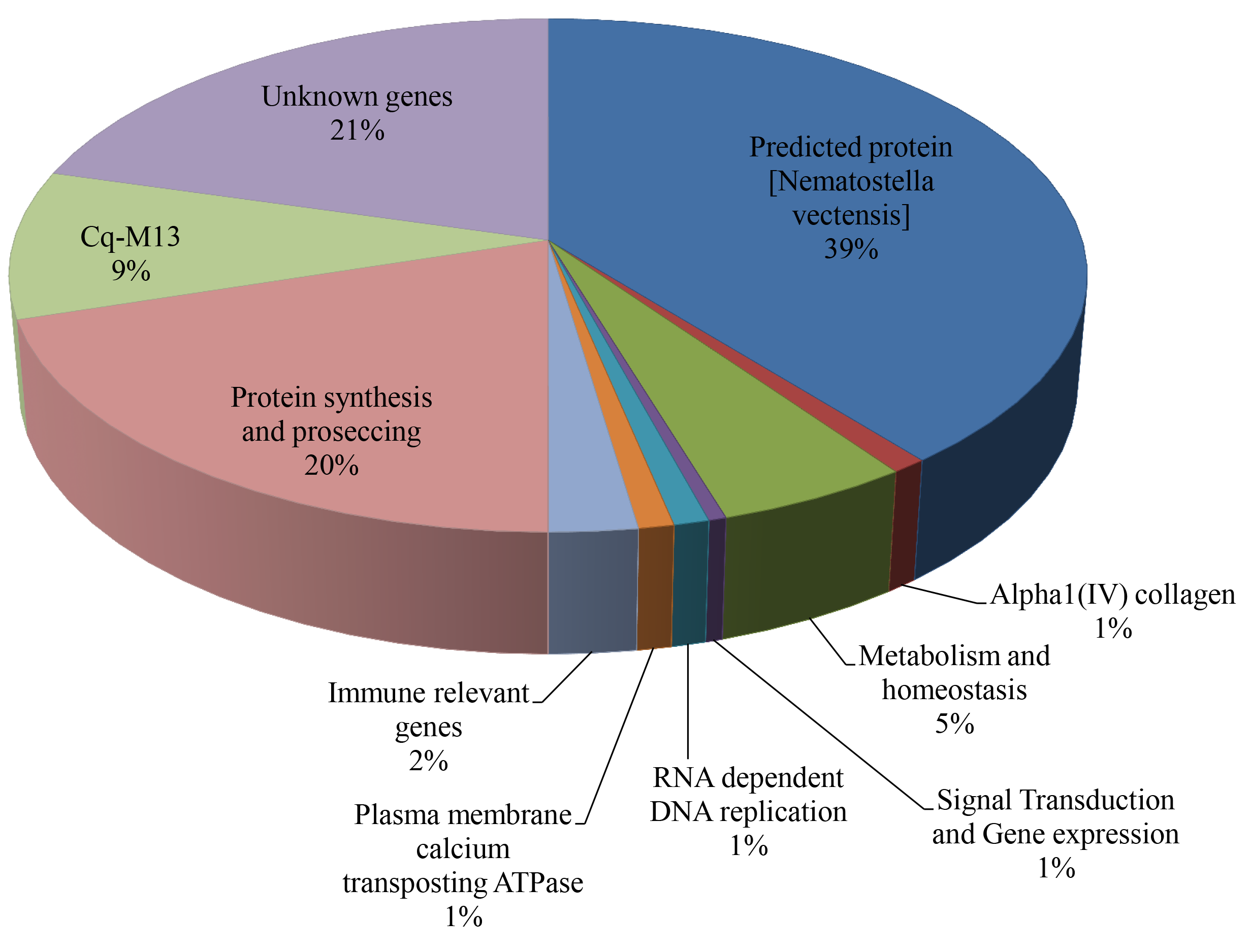

Supplement: S1 Fig — More than 21% of the sequences had no significant similarity (E-value > 0.01) to any Uniprot protein. 39% were similar to hypothetical protein of sea anemone N. vectensis, 9% were similar to Cq-M13, and the rest could be related to proteins associated with the indicated GO categories. (TIF) [file pone.0127871.s001.tif]
